# Remarks on Diffusion as a Therapeutic Resource

**Published:** 1842-02-05

**Authors:** Charles B. Voight


### MEDICAL EXAMINER.

NEW SERIES.

No. 6.] PHILADELPHIA, FEB. 5, 1842. [Vol. I.

### ORIGINAL COMMUNICATIONS.

*Remarks on Diffusion as a Therapeutic resource.* By Charles B. Voight, M. D.

The ensuing observations are respectfully proposed to attract a more special attention to the principle of diffusion in the treatment of disease than it appears in general to command. The topic, it is conceded, is not new; and the fact affords encouragement in the prosecution of the present design. Ten years ago, when I was a member of the medical class in the University of Pennsylvania, it was forcibly inculcated from the chair of the Practice. With the writer it has become a favourite practical principle. I believe I can safely affirm that the whole tenor of my medical observations to the present time supplies me with accumulated evidence of its safety, utility, and value, when applied with the ordinary care of professional discrimination and judgment. As estimated by the writer, the merits of the subject would authorize an extended and formal essay, but for the sake of brevity and convenience, it is submitted in the form of a series of grouped and cursory observations.

I. By diffusion, I mean a centrifugal determination, or a general capillary efflux in a direction from the axis towards the periphery of the system. Its existence in a state of health is apparent and appreciable in the fulness, plumpness, succulency, and healthy hue and temperature of the body. By the pallor, the contracted and shrunken state of the lineaments, and coldness of surface, it is broadly distinguished from the centripetal concentrations of congestion. If the latter species of action be admitted, and it is universally recognized, the former cannot be denied. It is the object which is designed and accomplished in the cure of congestion. The one kind of action is the inversion of, and implies, the other. They are mutually correlative. It is a healthy action. All the indications and attributes of health are attendant upon it. It differs from fever as a normal and regular medium of action differs from a morbid, perverted, and complicated excess of action. In a therapeutic point of view, it mainly contemplates and consists in the establishment of a just relation between the blood and the capillary section of the circulatory apparatus; and so long as this relation is maintained the occurrence of vascular disease is, I believe, impossible.

In irritation, inflammation, and congestion, normal or physiological diffusion is subverted, and a new condition introduced. An undue proportion of blood is precipitated upon a particular part or parts, organs or tissues, and as no compensating supply is simultaneously acquired, it is obvious that the entire portion of the capillary system which, is unincluded in the disease, is deprived of its normal supply of blood to a degree commensurate with the morbid excess of the local accumulation. *Ubi irritatio ibi affluxus*,—an aphorism no less true at the present date than when first announced, and an example of the "perennity" of natural science. Now, diffusion meditates the restoration of the normal condition, by effecting an equable distribution of the blood over the system at large; detracting from local excess by instituting an equilibrium or normal relation of supply in the entire capillary system.

Is it not a healthy diffusion which supports a vigorous nutrition, and by which fattening or corpulency naturally takes place? If so, diffusion is a physiological law. Is not this the principle which has been set at work when a chronic irritation has been cured by a sea voyage or a country residence? Is it not to the same principle that, in part at least, may be referred the *embonpoint* generally attendant upon the habitual use of opium?*—an article which, besides other properties, exerts a diffusible action in a high degree. And may it not be asserted that the alterative power of mercury also largely resides in its diffusibility?

II. However indispensable blood-letting may be in the treatment of disease, it is very certain that it seldom of itself, or unaided by other means, perfects the cure. Medicinal agents are generally resorted to and required for the eradication of disease and the re-establishment of health. The mere abstraction of blood cannot be relied upon in practice; and it is equally well known that urged beyond limits which even in the robust are soon arrived at, it is often not only abortive as respects the cure, but sometimes hazardous, or absolutely pernicious. Nor can the antiphlogistic system in its lengthened detail, or the reducing process, though its claims to an appropriate place in the category of medical resources cannot be impugned, be depended upon as the ultimate and essential curative procedure. There are necessary limits to its application which, if transgressed, compromise the life of the patient, without curing the disease. Disease also occurs under circumstances where a recourse to depletion and reduction, by the perils it would engender, is inadmissible. Such is the case when it happens in the exhausted, or extremely infirm. Depletion here only rivets the local disease; and should the part affected involve a capital function, hurries its career to a fatal issue. The inherent forces of the general capillary system, by which diffusion and derivation are secured, and which resist and countervail centripetal revulsion, once exhausted, an overwhelming reflux falls upon the suffering organ and annihilates the play of the functions.

But local disease cannot be resolved without the eventual occurrence, whether artificially or spontaneously induced, of diffusion or

* Its collateral property of blunting sensibility probably also conduces to the same end.

an equalization of capillary action. This is the radical and essential curative process. To it all other therapeutic operations are tributary or adjuvant. Thus, general excitement and vigor are diminished because they administer to local excitement: and local excitement is reduced that the organic force, or irritability of the capillary system at large, no longer overpowered by local action, may restore a salutary diffusion.* Diffusion, therefore, in vascular disease, is the ultimatum of medical effort. It is the essential and indispensable condition of its cure; and hence, in the majority of therapeutic problems, demands of the physician a paramount consideration.

III. Principles in general, though extensively and beneficially available in the production and promotion of good, cannot, when brought to light, spontaneously apply themselves for the accomplishment of these objects; nor do they disclose, of themselves, the rules to be observed in order to ensure their apposite and successful employment. To human effort, enterprize, ingenuity, and judgment, in their middle station between the principle, on the one hand, and the applications of which it is susceptible, on the other, is allotted the task of defining the one and achievng the other.

How can the principle of diffusion be successfully converted to the cure of disease? What conditions does its application presuppose and require, that, when brought to act upon the system, its operation may do good and not harm? Towards the elucidation of these points, a few brief and desultory remarks occur, which may at least contribute to the end in view. Extended medical erudition, conjoined with the most ample and matured experience, would alone suffice to do full justice to this branch of the subject.

As previously intimated, the writer believes that an application of the principle in question spontaneously offers itself to notice when a chronic disease or local irritation,—bronchial or gastro-enteric, for example,—is cured by a change from a city to a country residence. The case is a familiar one, and the fact it conveys, the occurrence of the cure, of indisputable authenticity. What is the character of the operations by which this revolution in the economy is effected? The solution of this question will aid the present investigation.

Simultaneously with the subsidence of the local irritation, which is commonly of slow and gradual progression,—a notable feature of the cure,—four obvious results of the restorative causes at work upon the system are plainly evinced: these are, 1. An acquisition of vigor. 2. Increased deposition upon the periphery. 3. An improved complexion and temperature of the surface. 4. A restoration of some one or more of the secretions previously perverted, obstructed, or annulled; particularly, of perspiration. Now all of the three results last specified are referrible to, and denote, an alteration in the capillary actions of the individual; and an accession of force to the impulse of peripheral determination. Universal diffusion, which is a capillary phenomenon, has been set up in the place of centripetal or local concentration; and

* Principles of Medicine. By S. Jackson, M. D. Philadelphia.

in the perfection of the cure, finally overcomes and eradicates it. The disease is resolved by the institution of a permanent "systemic" capillary derivation,—a collateral and most propitious and salutary result of the therapeutic principle under vindication.

The foregoing exposition, which is deemed to be sound, natural, and philosophical, suggests some practical considerations to direct and regulate the application of the principle of diffusion. These are complex, and refer alike to the qualities of the agent to be selected for its accomplishment, and the condition of the patient upon whom diffusion is designed to be impressed.

The medicine must be adapted by virtue of inherent affinity, equably and uniformly to augment capillary irritability as an integral and vital property of an entire and all-pervading tissue of the body; and to facilitate, harmonize, and promote the functions of secretion and exhalation. A stimulant, of course, covers the first indication, or the augmentation of action which is to be induced; but as some stimulants, in particular cases, will seriously interfere with the secretions, and would therefore violate some of the conditions which are essential to be observed in an accurate imitation of the natural process of cure, as exhibited in the precedent which has been quoted above, the article to be employed must necessarily be characterized by a suitable congruity or correspondence of action with the general flux of the secretions; or, what imports the same thing, it should be devoid of all tendency to impede them.

The employment of a stimulant, distinctively viewed as such, also furnishes some suggestions of a similar purport. These relate to the condition of the patient on the one hand; and on the other, to the collateral properties which most stimulants possess, in common with medicines in general, of exerting other actions upon the system independently and irrespective of the action desired.

In reference to the collateral properties alluded to, it may be observed that, in consequence of the complications which are liable to accompany disease, it is possible sometimes to convert them to useful purposes; but the contrary frequently obtains, and that in the production of diffusion they should be sedulously guarded against. In a suitable case for its exhibition as a diffusible, camphor might be advantageously employed in reference to its antispasmodic qualities, to tranquilize nervous turbulency, in the treatment of a case of pulmonary congestion; but should a meningeal or cerebral excitement or irritation also co-exist, the operation of its antispasmodic qualities would be wholly inadmissible. Instead of tranquilizing, it would exalt cerebral organic action, and, in the place of diffusing the blood, it would throw a preponderance of it into the capillaries of the brain. The consequences need not be detailed. Here is a conspicuous example of a collateral operation of a diffusible, and it conveys a relevant and practical maxim of extensive analogical importance.

As nearly allied to the foregoing topic, it may be observed that the same tenor of remark holds equally good in relation to the production of expectoration in many instances of bronchial irritation. When general capillary vigor is unimpaired, it may, perhaps, be advantageously promoted; but, as a general rule, at least in chronic bronchitis, I believe it to be a superfluous and prejudicial practice; and if diffusion be the fundamental condition of the cure of local disease, as argued in this dissertation, the active promotion of expectoration in the case supposed is unphilosophical. An active expectorant (some are passive in their operation) augments action, not upon or in a direction towards the periphery, but conversely near to and towards the axis of the system, which is an abnormal or morbid course of action. They should therefore be used in chronic bronchitis with much reserve, and when much general capillary inactivity marks the case, those of the active kind, or such as stimulate the bronchial vessels, should be entirely proscribed. These are absolute local stimuli, and tend to confirm the disease by inverting and contravening the recuperative action of diffusion, and aggravating excitement in the identical vessels already over excited as the special attribute of their pathological condition. An artificial augmentation of local vascular action is incompatible with the principle of diffusion.

The condition of the patient, in relation to the employment of stimuli, the topic next to be disposed of, may be sufficiently considered for the present purpose by contemplating it under the two-fold and opposite aspect of sthenic fever on the one hand, and general capillary feebleness on the other. The habitudes of the other states of system, in their various gradations between these extremes, will be sufficiently referred to incidentally, to be appreciated or perceived without a more detailed and special discussion. To examine them particularly is necessarily precluded by a due regard to reasonable limitations.

When sthenic febrile excitement complicates a local excitement, as commonly happens in individuals of considerable constitutional vigour, an equalization of the circulation cannot of course be attempted by the application of stimuli. Local excitement seldom or never abates prior to the reduction of general vigour and high excitement; on the contrary, they contribute to it. The first step universally assumed, under such circumstances, is depletion or reduction by either direct or indirect means. General excitement being first overcome, exaggerated topical action is quelled, and comes more easily under control. This is next reduced, in order to bring the forces at work in the diseased capillaries to a level with those which naturally reside in the general capillaries. This fully accomplished, resolution, in sound and tractible constitutions, will often ensue and effect the cure. But should they fail at this point to compass this salutary alteration in the circulation, a diffusible, peculiarly adjusted to the distinctive features of the condition in question, offers itself as the appropriate indication of cure; the object of which is to determine the predominance of action to the side of the capillaries at large. When, therefore, the active or direct application of the principle of diffusion in the treatment of diseases appertaining to the present condition is called for, the provision to be observed to secure its successful operation is a preliminary and suitable recourse to antiphlogistic measures.

The opposite of the preceding condition, or constitutional feebleness conjoined with general capillary inefficiency of action, offers an apposite case for the immediate appeal to the capillary stimulant. This is the condition which embraces the extensive array of chronic disease. The cases it presents are usually intractible to the antiphlogistic system of treatment. Reduction, it is true, by diminishing the topical excitement, may, and often will, afford some momentary alleviation; but as it fails to introduce an adequate diffusion, the relief is only temporary and delusive. But this is not all. The general capillaries, by participating in the loss of tone which reducing measures necessarily inflict, sustain a positive injury. They are still farther removed by it from the capacity of instituting a recuperative derivation. The local action marches onward to disorganization, and ultimately eventuates in a fatal lesion. Patients of this description, or such as are presented by the condition under review, furnish the examples of the spontaneous cures which are so frequently accomplished by travelling, or a change of residence. Diffusion, benignly and seasonably obtained in these cases, promptly and conspicuously displays a salutary amelioration. A new direction is imparted by it to the current of the circulation. The local raptus is subverted and merged in the general sanguine dispersion, which needs only a proper and steady support to revolutionize the system and reinstate the order of health.

The foregoing observations upon the application of this principle, may be summarily exhibited in the following recapitulation:

1. The diffusible agents to be employed should be characterized by an entire compatibility with the performance of the secretions.

2. The medicine in most cases should be an exclusively capillary stimulant; that is, totally destitute of collateral therapeutic qualities.

3. Should collateral actions be admissible and desirable, they should be subordinate to, and co-operate with, the main action of diffusion.

4. An augmentation of a central morbid action, or local raptus, whether incidentally or primarily induced, is a violation of the law of diffusion, and morbific.

5. General vigour and high febrile excitement impose the observance of a preliminary antiphlogistic practice, in order to equalize the local with the general capillary forces.

6. The prevalence of general capillary inertion, and the absence of constitutional activity and vigour, demand, in general, an immediate appeal to the principle of diffusion.

7. When constitutional feebleness and inactivity, and general capillary languor unite in the same case, depletion or reduction is detrimental. It exhausts the capillary forces of the system at large, whereby local excess of action is overcome.

A few remarks in reference to diffusible agents, will complete the scope of the present article. This property, as is well known, appertains to an extensive range of medicines. The arterial, nervous, and narcotic stimuli, and some of the diaphoretics, exert it in a marked degree, and consequently may be employed in reference to it; but, as pointedly indicated in a former place, the selection of the agent for clinical purposes must be guarded by a studious attention to collateral properties. These, with the most of them, are numerous and diversified; and hence arises a difficulty in the establishment of a salutary diffusion. As one step in the way of overcoming this impediment, and facilitating the application of the principle, the following combinations are offered as specimens of capillary stimulants.

When the case is distinguished by constitutional feebleness and general capillary languor, the carbonate of ammonia, in union with the gum guaiacum, will, in general, fully answer expectation. They are conveniently administered in mucilage, in the proportion of two scruples of each of the former articles, to four ounces of the latter. The dose a dessert spoonful every three or four hours. To meet *special* indications, I have sometimes improved this medicine by the addition of small quantities of the Pulv. Ipecac., or Hoffman's Anodyne.

Should the carbonate of ammonia be contra-indicated by a considerable degree of constitutional activity and a febrile tendency, it may be replaced by the nitrate of potassa, or the muriate of ammonia; but as these articles are powerfully antiphlogistic or reducing, and therefore tend to depress general capillary power, much circumspection is required in the adjustment of their relative proportions. Constitutional and local activity should be restrained by them, while diffusion is insured by an adequate impression of the guaiacum.

The subject is submitted to professional attention. Should the remarks which it has suggested impart to it such favour of acceptance as will obtain for it clinical examination or trial, the object of the writer will be gained. Little more has been attempted than a mere statement and general exposition of it. Beyond this it is commended to farther investigation. Familiar and habitual reference to it in practice has inspired a steady conviction of its value, and justifies and encourages the belief that a similar result will attend its further application.

