# Erratum in No. 6

**Published:** 1842-02-12

**Authors:** 


*Erratum in No. 6.*—In Dr. Voight's paper, p. 86, l. 6, for, "This is the condition which embraces the extensive array of chronic disease," read, "this condition embraces *an* extensive array of chronic disease."

